# Nephroblastoma Arising from Primary Testicular Germ Cell Tumor: A Case Report and Literature Review

**DOI:** 10.1155/2016/7318672

**Published:** 2016-11-10

**Authors:** Houda Alatassi, Brittany E. O'Bryan, Jamie C. Messer, Zhenglong Wang

**Affiliations:** ^1^Department of Pathology and Laboratory Medicine, University of Louisville School of Medicine, Louisville, KY, USA; ^2^Department of Urology, University of Louisville School of Medicine, Louisville, KY, USA

## Abstract

Adult extrarenal nephroblastoma is a very rare tumor. Nephroblastoma arising from primary testicular germ cell tumor is exceedingly rare. To our knowledge, only three cases have been reported in the English literature. We report a case of a 19-year-old man who presented with a large right testicle. Image studies showed a large retroperitoneal mass along with liver and lung metastases. Orchiectomy demonstrated a mixed germ cell tumor composed of yolk sac tumor, embryonal carcinoma, and mature and immature teratoma with a significant portion of nephroblastoma. The patient received chemotherapy and no recurrence was noted during six months of followup. WT-1 expression was also studied due to the lack of consistency of its expression in testicular nephroblastoma in the literature. We also present a discussion and review of the literature due to its rarity, which indicate an adverse prognosis for patients with nephroblastoma components receiving standard chemotherapeutical regimes for testicular germ cell tumors.

## 1. Introduction

Although nephroblastoma is the most common primary renal malignant tumor in children, its occurrence at extrarenal sites in adults is very rare. The reported extrarenal locations included retroperitoneal, iliac, pelvic, and inguinal regions [1–4]. There are few reports of nephroblastoma arising from either primary or metastatic testicular germ cell tumors [5–7]. The presence of somatic components in testicular germ cell tumor, including nephroblastoma, may indicate resistance to chemotherapy [8]. Due to its rarity, there is no consensus regarding clinical management. In addition, our understanding of its histological origin and molecular mechanisms will depend on the accumulation of reported cases in the literature. Herein, we report a case of nephroblastoma arising in a primary testicular mixed germ cell tumor.

## 2. Case Report

**Figure 1:**
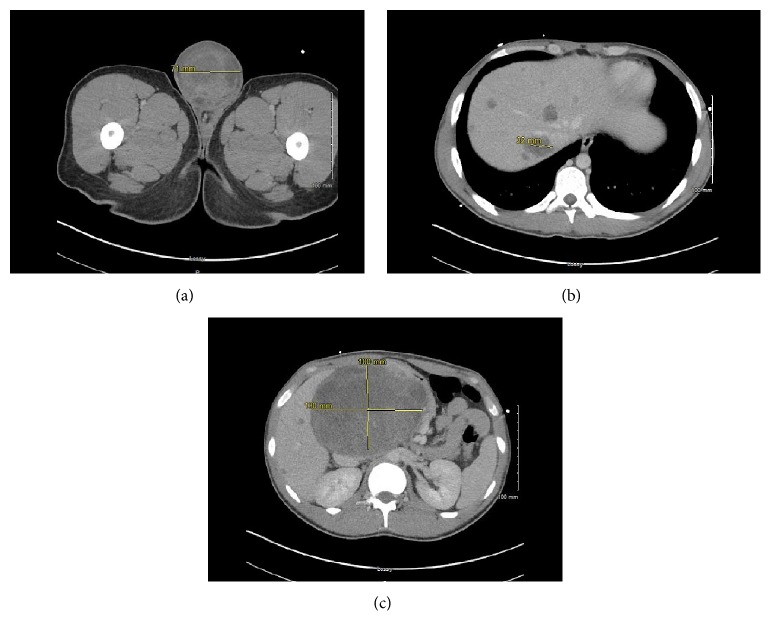
Pelvic computed tomography showing a 9 cm heterogenous mass in right testicle (a). Abdominal CT scan showed multiple hepatic masses (b) and one large heterogenous retroperitoneal mass (c) measuring up to 13 cm in greatest dimension, consistent with metastatic disease.

A 19-year-old, otherwise healthy male presented to the Emergency Department due to a syncopal episode. He was noted to have a recent history of lethargy and profound weight loss. Physical examination revealed a nontender, mobile firm right testicular mass. The patient confirmed a 5-month history of an enlarged swollen right scrotum. Pelvic computed tomography (CT) showed a 9 cm heterogeneous mass in the right testis (Figure 1(a)). Abdominal CT scan showed multiple hepatic masses and one large heterogenous retroperitoneal mass measuring up to 13 cm in greatest dimension, consistent with metastatic disease (Figures 1(b) and 1(c)). Chest CT was remarkable for multiple pulmonary nodules measuring up to 2.1 cm in greatest dimension. Laboratory studies showed elevated serum human chorionic gonadotropin (hCG; 53809.0 mIU/mL) and alpha fetal protein (AFP; 6630.0 ng/mL).

**Figure 2:**
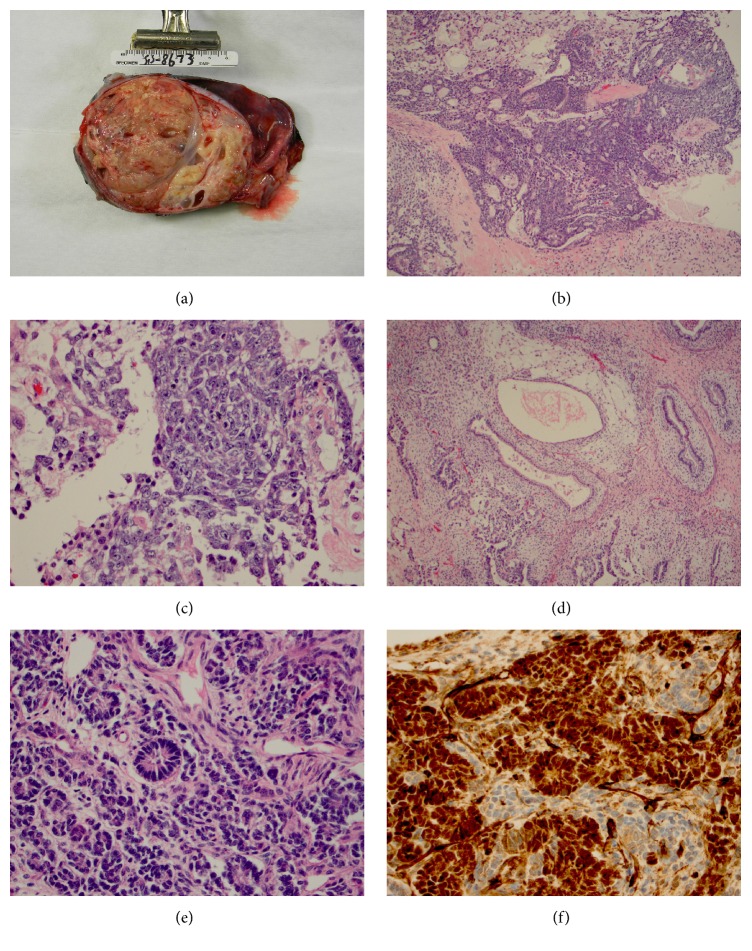
(a) Encapsulated solid mass with interspersed cystic areas. Yolk sac tumor component (b), embryonal carcinoma component (c), and teratoma component (d) were identified. (e) Nephroblastoma component shows typical blastema and epithelial components. (f) Nephroblastoma component has strong WT-1 expression.

With the clinical impression of a testicular neoplasm, the patient was taken to the operating room for a right radical orchiectomy. Gross examination of the orchiectomy specimen demonstrated a 10 cm encapsulated solid mass with interspersed cystic areas replacing the entire testicular parenchyma. The cut surface of the tumor was soft and tan-brown to yellow in color (Figure 2(a)). Microscopically, the testicular tumor was composed of immature teratoma with nephroblastoma (40%), mature teratoma, embryonal carcinoma and yolk sac tumor (Figures 2(b)–2(d)). The nephroblastoma component consisted of undifferentiated blastema, fibroblast-like stroma and epithelial elements (Figure 2(e)). Immunohistochemical stain for WT-1 was strongly positive in the nephroblastoma component only (Figure 2(f)).

The patient's serum hCG and AFP remained elevated until after completing chemotherapy. He received six cycles of VP-16, ifosfamide, and cisplatin (VIP). Postchemotherapy imaging studies showed partial response of the hepatic and pulmonary visceral metastases with persistence of the large retroperitoneal mass.

## 3. Discussion

Although nephroblastoma or Wilms tumor is the most common malignant renal tumor of children occurring between 2 and 5 years of age, extrarenal nephroblastomas in adults are very rare and most are reported after chemotherapy.

On the other hand, germ cell tumors are the most common tumor in young men. Somatic type malignant components in germ cell tumors are occasionally observed [9–12]. Nephroblastoma arising from primary testicular germ cell tumor is exceedingly rare. To our knowledge, only three cases have been reported in the English literature. Emerson et al. reported a case of malignant testicular mixed germ cell tumor in a 22-year-old patient with nephroblastoma and rhabdomyosarcoma components [13]. Interestingly, WT-1 was negative in this case. Keskin et al. reported a case of a 19-year-old man with nephroblastoma being the only nongerm cell component [14]. WT-1 staining was focally positive. Vanasupa et al. reported a nephroblastoma arising from an atrophic testis [15]. In addition, nephroblastoma differentiation has been previously reported in metastatic teratomas [5–7]. Although the presence of other somatic components in germ cell tumors indicates resistance to chemotherapy for these tumors, the clinical significance of nephroblastoma is still unknown. The patient reported by Emerson et al. developed supraclavicular and retroperitoneal metastatic teratomas after chemotherapy [13]. Our patient only showed partial responses to the chemotherapy. These observations may indicate an adverse prognosis for patients with nephroblastoma components receiving standard chemotherapeutical regimes for testicular germ cell tumors. More case reporting and studies will be needed to address the clinical significance of nephroblastoma arising from testicular germ cell tumor.

Although WT-1 mutation was well documented in renal nephroblastoma, the histological origin and molecular mechanisms of testicular nephroblastoma are poorly understood. The potential histology origins include Sertoli cells and rete testis, which have retained WT-1 expression in adult testis. WT-1 expression was observed in two of the three reported cases [13–15]. One case showed diffuse positivity and one had focal positive staining. The third case, despite the lack of WT-1 expression in the nephroblastoma component, demonstrated a common clonal origin for nephroblastoma and other germ cell tumor components in testis. For our case, we compared the staining of WT-1 in nephroblastoma and germ cell components. WT-1 expression was strong and diffusely positive in the nephroblastoma component, whereas the germ cell components were negative. With known importance of WT1 gene in renal nephroblastoma, the different staining patterns of WT-1 in nephroblastoma and germ cell components may suggest a potential similar role of WT1 gene activation in the development of nephroblastoma in testicular germ cell tumors. Future case reporting and studies will be needed to confirm its importance due to the rarity of these cases.
